# A novel PAX3 mutation in a Korean patient with Waardenburg syndrome type 1 and unilateral branch retinal vein and artery occlusion: a case report

**DOI:** 10.1186/s12886-018-0933-9

**Published:** 2018-10-11

**Authors:** Eun Young Choi, Wungrak Choi, Christopher Seungkyu Lee

**Affiliations:** 1 Department of Ophthalmology, The Institute of Vision Research, Gangnam Severance Hospital, Yonsei University College of Medicine, 211, Eonjuro, Gangnam-gu, Seoul, 06273 Korea; 2 Department of Ophthalmology, The Institute of Vision Research, Severance Hospital, Yonsei University College of Medicine, 50-1, Yonseiro, Seodaemun-gu, Seoul, 03722 Korea

**Keywords:** Waardenburg syndrome, PAX3 gene mutation, Hyperhomocysteinemia, Branch retinal vein occlusion, Branch retinal artery occlusion

## Abstract

**Background:**

Waardenburg syndrome (WS) is a very rare genetic disorder affecting the neural crest cells. Coexistence of branch retinal vein occlusion (BRVO) and branch retinal artery occlusion (BRAO) in the same eye is also a rare finding. Here we report a case of WS type 1 that was confirmed by a novel mutation with the finding of unilateral BRVO and BRAO.

**Case presentation:**

A 36-year-old, white-haired Korean man presented with a complaint of loss of vision in the inferior visual field of his right eye and hearing loss. He had telecanthus with a medial eyebrow and a hypochromic left iris. Funduscopy showed an ischemic change at the posterior pole in the right eye with sparing of the foveal center as well as retinal hemorrhages and white patches along the superotemporal arcade. Fundus angiography revealed the presence of both BRVO and BRAO, and optical coherence tomography showed thickening and opacification of the retinal layers corresponding to the ischemic area. A blood workup revealed hyperhomocysteinemia and the presence of antiphospholipid antibodies; both are suggestive as the cause of the BRVO and BRAO. Single nucleotide polymorphism analysis confirmed a novel *PAX3* mutation at 2q35 (c.91–95 ACTCC deletion causing a frameshift). These findings confirmed a diagnosis of WS type 1.

**Conclusions:**

WS is a heterogeneous inherited disorder of the neural crest cells that causes pigment abnormalities and sensorineural hearing loss. This is the first report of unilateral BRVO and BRAO in a patient with WS. Furthermore, the *PAX3* mutation identified in this patient has not been reported previously.

## Background

Waardenburg syndrome (WS) is a rare heterogeneous inherited disorder of the neural crest cells (NCC) [1, 2] that causes abnormalities in NCC-derived melanocytes, leading to pigment abnormalities and sensorineural hearing loss. Read and Newton identified four types of WS according to the additional symptoms present [3]. In their classification, type 1 (OMIM #193500) and type 2 (OMIM #193510) have similar features, but are distinguished by telecanthus, which is present only in type 1. Musculoskeletal anomalies are found in type 3 (OMIM#148820), while type 4 (OMIM #277580) is associated with Hirschsprung disease. At the molecular level, six genes are involved in this syndrome and have varying degrees of frequency: *PAX3* (encoding the paired box 3 transcription factor) is associated with types 1 and 3, *MITF* (microphthalmia-associated transcription factor) and *SNAI2* (snail homolog 2) with type 2, *EDN3* (endothelin 3) and *EDNRB* (endothelin receptor type B) with type 4, and *SOX10* (Sry bOX10 transcription factor) with types 2 and 4 [4].

Coexistence of branch retinal vein occlusion (BRVO) and branch retinal artery occlusion (BRAO) in the same eye is a rare finding. Lee et al. reported 56 cases of coexisting arterial insufficiency in a study of 308 eyes with BRVO [5]. Subsequently, case series of patients with both BRAO and BRVO and comorbid systemic associations were reported [6, 7]. Commonly associated systemic comorbidities included multiple cardiovascular risk factors, cardiac valve disease, hyperhomocysteinemia, a hypercoagulable state, systemic lupus, and vasculitis. Therefore, systemic evaluation is routinely recommended in addition to appropriate treatment in these patients. Coexisting BRVO and BRAO has not been reported in a patient with WS.

Here we describe a patient with WS type 1 who was found to have unilateral BRVO and BRAO and in whom single nucleotide polymorphism analysis identified a novel *PAX3* mutation in the 2q35 region.

## Case presentation

**Fig. 1:**
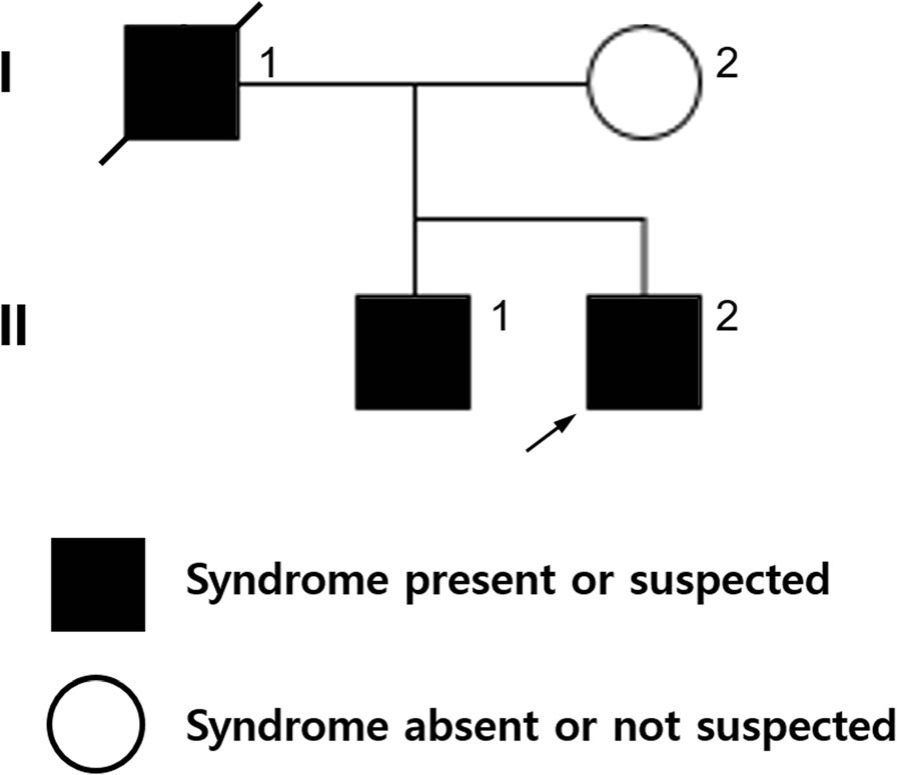
Pedigree of a family with Waardenburg syndrome. The arrow indicates the proband (II-2). The square indicates male sex and the circle indicates female sex. The solid symbol represents a family member with hearing loss and the clear symbol indicates a family member without hearing loss. The symbol with a diagonal line indicates a deceased family member

**Fig. 2:**
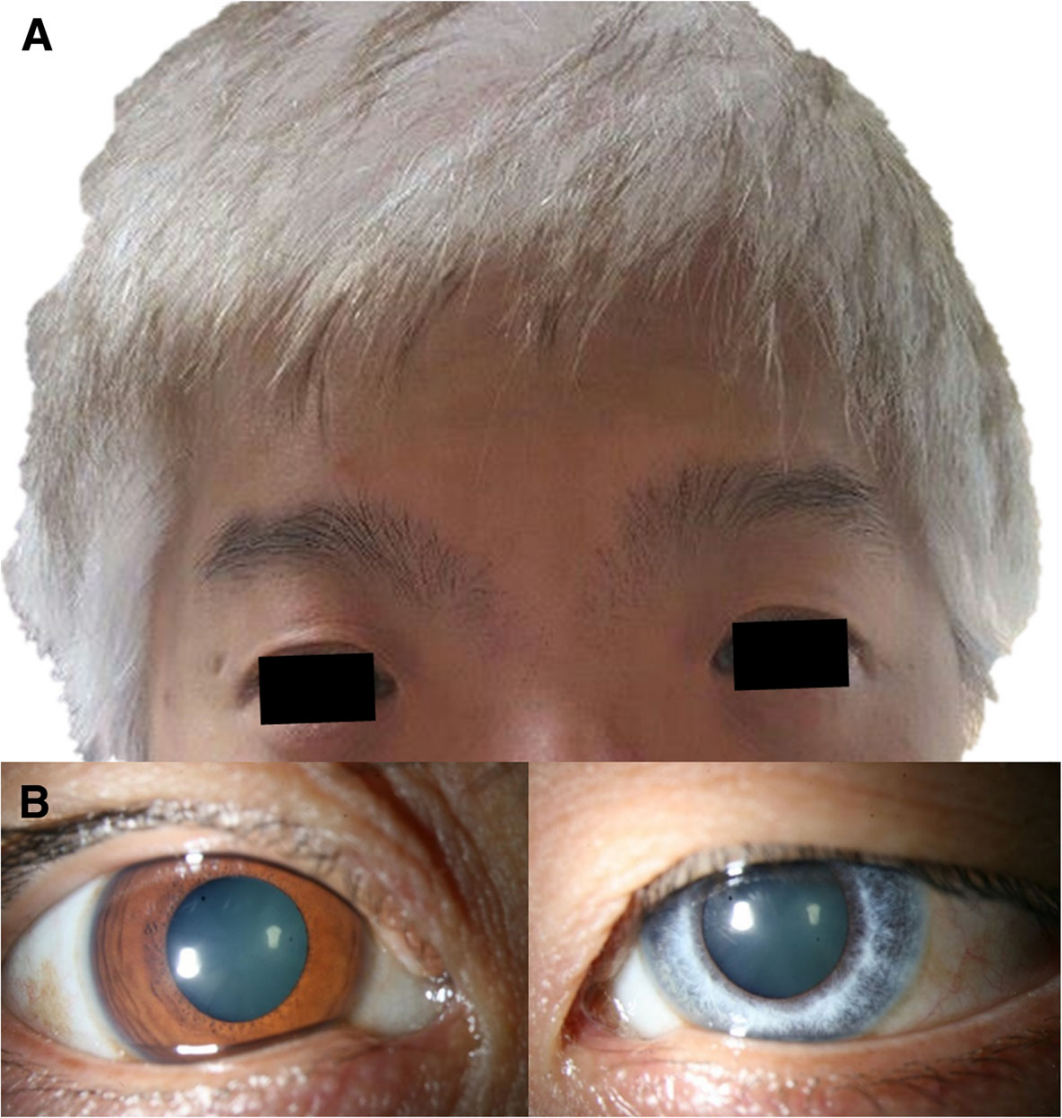
The proband is a 37-year-old man with white hair, dystopia canthorum with a medial eyebrow (**a**), and a gray iris in the left eye (**b**)

**Fig. 3:**
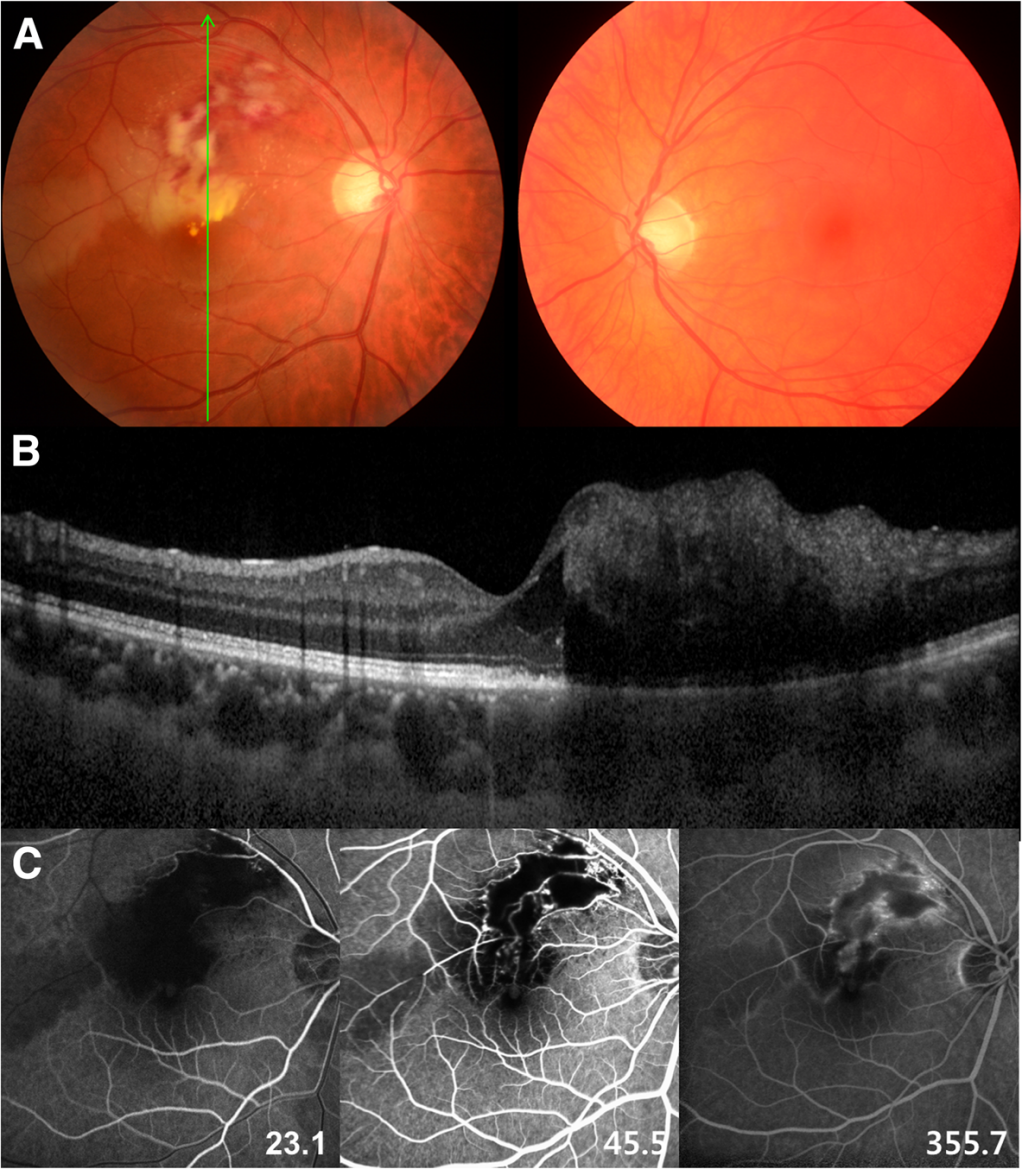
**a** The right fundus, showing an ischemic change at the posterior pole with foveal sparing as well as retinal hemorrhages and white patches along the superotemporal arcade. Choroidal hypopigmentation is visible in the left fundus. **b** An optical coherence tomography image showing thickening and opacification of the retinal layers corresponding to the ischemic area. **c** On fundus angiography (FA), a nonperfused area is seen superior to the macula because of the coexistence of branch retinal vein and artery occlusion. FA of the right eye shows a significant filling delay of the branches of the superotemporal retinal artery with a corresponding ischemic area (23.1 s). Sludging of the retinal artery is apparent and the retinal vein branches are tortuous and dilated (45.5 s). Capillary non-perfusion in the circulation of the superotemporal vein is observed. The perfusion defect is still present with the absence of superotemporal retinal artery branches (355.7 s). Neovascularization is not observed

A 36-year-old, white-haired Korean man (Fig. 1. II-2: Proband) visited the ophthalmology department complaining of loss of vision in the inferior visual field of his right eye. His face was characterized by lateral displacement of the inner canthus of both eyes with a medial eyebrow and a high broad nasal bridge (Fig. 2a). His medical history was significant for paralysis of one arm after a cerebral infarction 13 years earlier and right-sided sensorineural hearing loss. His father (Fig. 1. I-1), who had had hearing impairment, died of a myocardial infarction in his 50s, and his brother (Fig. 1. II-1) had bilateral hearing loss and heterochromia iridis. His best corrected vision was 20/25 with myopic correction (− 2.50 diopters) on the right and 20/20 with myopic correction (− 3.50 diopters) on the left. His intraocular pressure was 15 mmHg in the right eye and 13 mmHg in the left eye. A hypochromic left iris (Fig. 2b) was observed on slit-lamp examination. Funduscopy showed an ischemic change at the posterior pole with sparing of the foveal center along with retinal hemorrhages and white patches along the superotemporal arcade (Fig. 3a). Optical coherence tomography revealed thickening and opacification of the retinal layers corresponding to the ischemic area (Fig. 3b). Both BRVO and BRAO were detected on fluorescein angiography (Fig. 3c). An intravitreal anti-vascular endothelial growth factor (Avastin®, bevacizumab) injection (1.25 mg in 0.05 mL) was administered in the right eye for macular edema. After 2 months, the patient’s macular edema was significantly improved and his visual acuity was maintained at 20/25.

**Fig. 4:**
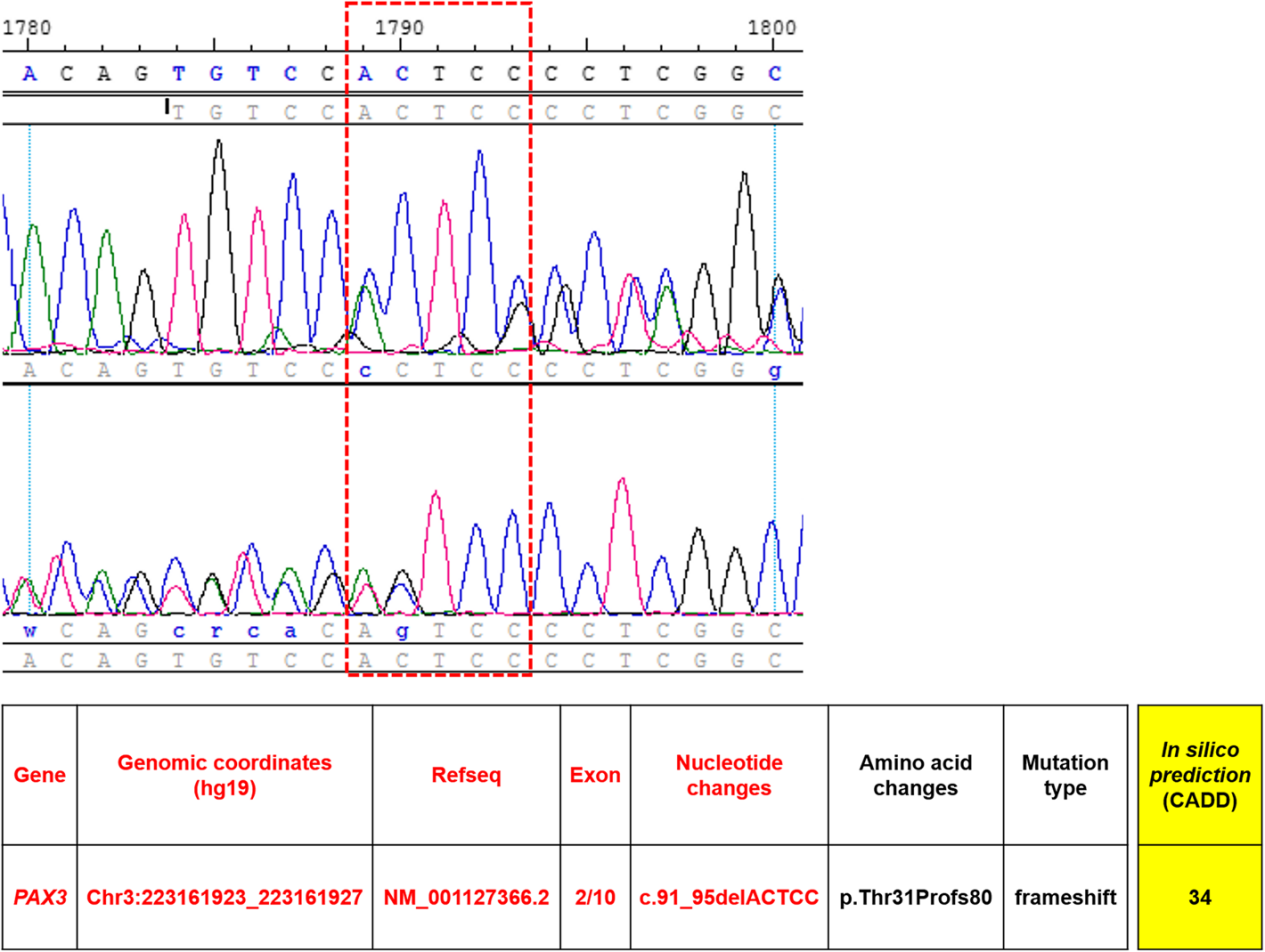
Results of single nucleotide polymorphism (SNP) analysis revealed a *PAX3* mutation in exon 2 on chromosome 2q35. An ACTCC deletion at c.91–95 caused a frameshift of protein Thr31. The SNP analysis was carried out according to the recommendations of the manufacturer (Applied Biosystems, CA, USA). Polymerase chain reaction products were prepared after optimization, amplification, and purification processes. The 96-capillary 3730xl DNA Analyzer (Applied Biosystems) was used for high-throughput SNP mapping and discovery. Combined Annotation Dependent Depletion (CADD) v1.4 was used to predict the pathogenicity of variants. The identified novel mutation was highly deleterious for *PAX3* (CADD score = 34)

Single nucleotide polymorphism analysis was performed by comparing a peripheral blood sample with the NM_181457 reference, and a *PAX3* mutation was confirmed in exon 2 on chromosome 2q35 (Fig. 4). An ACTCC deletion was noted at c.91–95, causing a frameshift of protein Thr31. A diagnosis of WS type 1 was made on the basis of this genetic mutation and the patient’s clinical features. Further biochemical workup revealed a raised serum homocysteine level (28.3 μmol/L). Antiphospholipid antibodies, including anticardiolipin and lupus anticoagulant, were also detected. Other blood coagulation factors were in the normal range or negative. The electrocardiogram showed no significant abnormality.

## Discussion

WS was initially described as an autosomal dominant disorder associated with depigmentation abnormalities and sensorineural hearing loss [1, 2, 4]. Other clinical manifestations, including dysmorphic craniofacial features [2, 8], upper limb abnormalities [2, 9], Hirschsprung disease [3, 10], and neurologic defects [11], have been reported with variable frequency. WS is now known to consist of a group of genetically heterogenous subtypes, and not all cases are inherited in a dominant manner. Given that the syndrome is caused by a genetic disorder affecting NCCs, the melanocytes of the skin and inner ear, peripheral and enteric nervous systems, and some of the craniofacial and skeletal tissues can in theory be affected [12, 13]. All six genes (*PAX3, MITF, EDN3, EDNRB, SOX10,* and *SNAI2*) known to cause WS [4] are involved in a complex interplay relating to the differentiation and function of melanocytes. Heterozygous mutations in *PAX3* are known to be responsible for most cases of WS types 1 and 3.

In this case, we identified a novel *PAX3* mutation (c.91–95 ACTCC deletion causing p.Thr31fs) in exon 2, which has not been reported previously. The pathogenicity of this frameshift mutation was validated by in silico prediction using Combined Annotation Dependent Depletion (CADD) version 1.4 [14]. The mutation was predicted to be highly deleterious for *PAX3* (CADD score = 34). It is well known that *PAX3* mutation is likely to cause WS type 1 [15–22]. The clinical characteristics in our patient supported a diagnosis of WS type 1, i.e., pigment abnormalities of the hair and left eye, congenital hearing loss, and dystopia canthorum. No musculoskeletal, neurologic, or intestinal anomalies were detected. The main limitation of this report is that the underlying pattern of inheritance of WS in the patient’s family could not be investigated. However, we constructed the family pedigree based on the hearing-impaired phenotype, which suggested that the de novo mutation (c.91-95delACTCC) identified in *PAX3* was the likely cause of genetic transmission of hearing loss in the patient’s family. In addition to family genotyping, further studies that include functional analysis are required to explore the genetic mechanism of this novel mutation.

It is of interest that our patient had both BRVO with BRAO in the eye contralateral to the depigmented left eye. Although the relationship is not clear, the angiographic findings suggest that the initiating event was retinal vein occlusion followed by stasis of blood flow. An elevation of intraluminal capillary pressure caused by a patent central retinal artery seemed to result in BRAO in the same quadrant as the BRVO. Considering the favorable visual outcome in this patient after 2 months of follow-up, arterial insufficiency rather than frank obstruction seems more likely. Laboratory investigations identified hyperhomocysteinemia [23, 24] and antiphospholipid antibodies [25, 26] as the possible cause of the unilateral BRVO and BRAO in this young patient. Kadoi et al. have previously reported BRVO of a hypochromic eye in a patient with WS that was considered most likely to be caused by systemic hypertension [27].

In vitro and animal studies have shown that the homocysteine level in the embryonic stage affects the expression of *PAX* [28, 29]. The relationship between homocysteine and *PAX3* mutation in WS is unclear. However, it is possible that the homocysteine level could be an environmental factor contributing to the variable clinical expression and familial penetrance of phenotypes in WS.

## Conclusion

We have encountered a young male patient who presented with a unilateral visual field defect associated with both BRVO and BRAO in his right eye. The diagnosis of WS type 1 was confirmed by his characteristic clinical features and detection of a novel *PAX3* mutation at 2q35 (c.91–95 ACTCC deletion). This report is the first to describe coexistence of BRVO and BRAO in association with hyperhomocysteinemia and antiphospholipid antibodies in a patient with WS. Further studies are needed to identify the association between WD and hyperhomocysteinemia.

## Abbreviations

BRAO: Branch retinal artery occlusion
BRVO: Branch retinal vein occlusion
EDN3: Endothelin 3
EDNRB: Endothelin receptor type B
MITF: (microphthalmia-associated transcription factor)
PAX3: (encoding the paired box 3 transcription factor)
SNAI2: (snail homolog 2)
SOX10: (encoding the Sry bOX10 transcription factor)
WS: Waardenburg syndrome
